# Treatment Satisfaction, Patient Preferences, and the Impact of Suboptimal Disease Control in Rheumatoid Arthritis Patients in Greece: Analysis of the Greek Cohort of SENSE study

**DOI:** 10.31138/mjr.33.1.14

**Published:** 2022-03-31

**Authors:** Prodromos Sidiropoulos, Andreas Bounas, Nikolaos Galanopoulos, Georgios Vosvotekas, Eftichia Maria Koukli, Panagiotis Georgiou, Nikolaos Marketos, Tina Antachopoulou, Antonios Kyriakakis, Maria Koronaiou

**Affiliations:** 1 Rheumatology, Clinical Immunology and Allergy, Medical School, University of Crete, Heraklion, Greece;; 2 OLYMPION Hospital-General Clinic of Patras, Patras, Greece;; 3 Outpatient Department of Rheumatology, University General Hospital of Alexandroupolis, Thrace, Greece;; 4 EUROMEDICA General Clinic of Thessaloniki, Thessaloniki, Greece;; 5 IASIO Hospital-General Clinic of Kallithea, Athens, Greece;; 6 Department of Rheumatology, General Hospital of Patras “Agios Andreas”, Patras, Greece;; 7 Private Clinic Henry Dunant Hospital Centre, Athens, Greece;; 8 Medical Department, AbbVie Pharmaceuticals S.A., Neo Iraklio, Athens, Greece

**Keywords:** rheumatoid arthritis, DMARDs, adherence, satisfaction, patient-reported outcomes, patient perspectives

## Abstract

**Objectives:**

SENSE was an international, non-interventional cross-sectional study that assessed treatment satisfaction in patients with suboptimally controlled active rheumatoid arthritis (RA) who were under treatment with any approved agent exposed to ≤ 2 biological disease-modifying anti-rheumatic drugs (DMARDs) at the time of enrolment. The current publication concerns the subanalysis of the results from the Greek cohort.

**Methods:**

Treatment satisfaction was assessed with Treatment Satisfaction Questionnaire for Medication (TSQM), with good treatment satisfaction defined as TSQM global ≥80. Adherence to therapy was recorded on a visual analogue scale (VAS) and treatment expectations were assessed on a 7-point numerical rating scale.

**Results:**

Of 121 patients, 82.6% were women, of mean age 64.8 years and mean time from diagnosis 8.4 years. Patients had active disease (mean DAS28-ESR 4.5) and compromised functional status (mean [SD] HAQ-DI 1.1 [0.7]) while on treatment (43.8% on biologics and 5% on steroids). The mean TSQM global was 66.9. Treatment expectations were “general improvement of arthritis” and “less joint pain” (mean score [SD], 4.9 [1.8] each), “more joint flexibility” (4.8 [1.9]), and “lasting relief of RA symptoms” (4.8 [2.1]). Oral administration was preferred by 65.3% of patients. Good self-reported adherence (≥80%) was recorded in 93.4% of the patients. Treatment switch to another DMARD was planned by treating rheumatologist for only 49.6% of the participants, despite suboptimal RA control.

**Conclusion:**

Patients with suboptimally controlled RA in Greece have low treatment satisfaction and poor self-reported outcomes, albeit high self-reported treatment adherence. Similarly to the global SENSE study results, the need for patient-centric treatment approaches in order to improve disease outcomes is emphasised.

## INTRODUCTION

Rheumatoid arthritis (RA) is a chronic, immune-mediated, inflammatory disease that, if not properly controlled, may result in progressive articular damage, loss of function, compromised quality of life, and increased mortality.^1^ Two types of disease-modifying antirheumatic drugs (DMARDs), biological DMARDs (bDMARDs), and targeted synthetic DMARDs (tsDMARDs), are therapeutic options for patients with inadequate response to conventional synthetic DMARDs (csDMARDs) that are recommended by the European League Against Rheumatism (EULAR) for the management of RA.^2^ Although the number of treatment options is steadily increasing and different drug classes have managed to slow down disease progression, many RA patients remain suboptimally controlled^3^ and sustained remission is rarely achieved.^3–9^

It has been shown that patients who do not achieve treatment targets have worse short- and long-term outcomes and timely treatment adjustments according to treat-to-target (T2T) principles, considering patient preferences and perspectives are critical to prevent disability.^9–12^ Although patients’ perspectives are important determinants of treatment success in RA, they have not been adequately evaluated. Most of the studies on RA have focused on outcomes reported by the treating rheumatologists. Databases worldwide and local registries have contributed information on RA patients’ and disease characteristics, including standard of care. Nevertheless, the evaluation of RA patients’ preferences, expectations, and self-reported outcomes, such as adherence to treatment, particularly in suboptimally controlled patients with active disease, including patients with moderate to high disease activity despite treatment with DMARDs, can contribute valuable insights on potentially unmet needs and maximize treatment benefits.^13–15^ Satisfaction correlates with patients’ treatment expectations, which can differ from rheumatologists’ treatment goals,^16^ and is in turn linked to patient treatment adherence.^13,17,18^ Increasing evidence suggests that adherence to RA treatment can be improved via patient support programs (PSPs)^19–21^ and patient empowerment via access to digital health-related information for informed decision-making.^22–24^ The effectiveness of the latter is related with the patients’ digital health literacy (DHL), ie, the ability to access and use credible online health information.

The international non-interventional cross-sectional SENSE study was conducted in 18 countries worldwide between September 2018 and May 2019 to determine the impact of inadequate response to DMARDs on treatment satisfaction and various disease outcomes and to analyse patients’ attitudes and perspectives toward treatment and their disease.^15^ SENSE also provided an opportunity to assess DHL in a large multinational cohort of patients with RA. In Greece, local RA databases, including the Hellenic Registry of Biologic Therapies, the nation-wide e-prescription platform, and the more recent country-wide database created by the RA Working Group of the Hellenic Rheumatology Society, have contributed information on RA and afflicted patient characteristics.^25–33^ Here, we report a sub-analysis of the global SENSE results from the patients that have been enrolled in seven rheumatology centres (public and private hospitals) in Greece (**Supplementary Table 1**).

**Table 1.** Sociodemographic characteristics.

**Characteristic**	**Patients, n ** **N=121**
Sex, female	100 (82.6)
Age, years, mean (SD)	64.8 (13.9)
Race	
White	121 (100)
Occupation	
Employed full-time	20 (16.5)
Employed part-time	
Unrelated to RA	2 (1.7)
Related to RA	3 (2.5)
Attending school or university	1 (0.8)
Unemployed	
Unrelated to RA	11 (9.1)
Related to RA	2 (1.7)
Early retirement	
Unrelated to RA	9 (7.4)
Related to RA	5 (4.1)
Regularly retired	69 (57.0)
Education	
No formal education	3 (2.5)
Primary school	28 (23.1)
Secondary school (e.g. high school)	65 (53.7)
Non-university, professional education	5 (4.1)
University	20 (16.5)
Residence	
Urban centre, population >80 000	49 (40.5)
Town, population 10 000–80 000	19 (15.7)
Rural area, population <10 000 inhabitants	53 (43.8)

All data are represented as n (%) unless otherwise stated.

RA, rheumatoid arthritis.

## MATERIALS AND METHODS

### Study design

The SENSE study was performed according to the Declaration of Helsinki with prior approval from each site’s Scientific Committee. Patient selection criteria included the following: Diagnosis of RA using either the 1987 revised American College of Rheumatology (ACR) or the 2010 ACR/EULAR classification criteria for RA; ongoing treatment with any approved csDMARD, tsDMARD, or bDMARD; and exposure to ≤2 bDMARDs at the time of the enrolment. All patients had to have residual disease activity as measured by Disease Activity Score, 28 joints (DAS28 >3.2) for 1 to <4 months before enrolment despite having received the full tolerable dose of current DMARD therapy for ≥3 months. Consecutive patients attending a routine rheumatologist office visit and fulfilling enrolment criteria were included in the study. Physicians collected data during a single scheduled visit.

### Assessments

Clinical parameters and socio-demographic characteristics were collected for all patients. Medical history including comorbidities (coded via the Medical Dictionary for Regulatory Activities system organ class level) and concurrent treatment, both for RA and overall were collected. Past medications for RA were also collected. Physicians were asked to report if switch to a different DMARD was planned for their patient, and the mode of action of planned treatment switches was captured.

The primary objective of the study was to assess patients’ treatment satisfaction related to current RA treatment using the Treatment Satisfaction Questionnaire for Medication, version 1.4 (TSQM v 1.4).^34^ This tool incorporates Effectiveness, Side Effects, Convenience, and Global Satisfaction domains, with scores ranging from 0 (poor satisfaction) to 100 (perfect satisfaction). Good treatment satisfaction is defined as TSQM global ≥80.^35^ VAS using numeric rating scales (NRS) were used to assess morning stiffness severity and duration (in minutes) as well as pain in the past 7 days (range 0 = “no stiffness/pain” to 10 = “worst possible stiffness/pain”).^15^ The following validated patient-reported outcomes (PROs) were used: Health Assessment Questionnaire – Disability Index (HAQ-DI) for physical function, Functional Assessment of Chronic Illness Therapy – Fatigue (FACIT-F) for fatigue, Work Productivity and Activity Impairment - Rheumatoid Arthritis (WPAI-RA) for workability, Short Form 36 Health Survey Questionnaire (SF-36) physical and mental component score for health-related quality of life (HRQoL).^36–39^ Self-reported adherence to medication was assessed using VAS, with good adherence defined as ≥80% on VAS.^40^ Patient medication preference information (PMPI), including preference for route of administration, combination therapy, time to effect, and acceptable side effects, was assessed by a 6-item questionnaire developed by AbbVie (**Supplementary Table 2**).^15,41,42^ Patient expectations for pharmacologic treatment were assessed using an 11-item questionnaire with a 7-point NRS (1 = “no improvement needed” to 7 = “the most improvement needed”). The need for PSP was assessed using a 17-item questionnaire with a 7-point NRS (1 = “not needed at all” to 7 = “very much needed”).

**Table 2.** RA disease characteristics.

Parameter, Score Range*	**Patients, n**	**Mean (SD)**
Time since RA diagnosis, years	121	8.4 (9.4)
TJC28, 0–28	121	7 (6.4)
SJC28, 0–28	121	3.4 (3.9)
PtGA, 0–10 cm	121	5.1 (1.9)
PGA, 0–10 cm	121	4.8 (1.7)
DAS28-CRP	107	4.2 (0.9)
DAS28-ESR	121	4.5 (1.0)
CDAI, 0–76	121	20.3 (10.1)
SDAI, 0–86	107	22.2 (10.7)

CDAI, Clinical Disease Activity Index; CRP, C-reactive protein; DAS28, Disease Activity Score, 28 joints; ESR, erythrocyte sedimentation rate; RA, rheumatoid arthritis; SDAI, Simplified Disease Activity Index; SJC28, swollen joint count based on a 28-joint assessment; TJC28, tender joint count based on a 28-joint assessment.

* Score are displayed to range from best health state to worst health state.

Healthcare resource utilization (HRU) during the three months before enrolment was also recorded and used to determine HRU over the past 12 months (by multiplying 3-previous month data with 4).

DHL was assessed by eHEALS, a self-report tool of 10 questions based on individuals’ perceptions of their skills and knowledge within each measured domain, providing scores ranging from 8 to 40; a higher total eHEALS indicates greater perceived skills at using online health information to help solve health problems; a score of <26 was considered to represent poor digital health literacy (DHL).^24,43^

### Statistical analysis

All statistical analyses were carried out using SAS^®^ software (version 9.4; SAS Institute, Cary, NC, USA). Quantitative data were described by the statistical parameters valid N, missing N, mean, standard deviation, median, minimum, maximum, lower quartile (25%), and upper quartile (75%). Qualitative data were described with (absolute and relative) frequency distributions. Two-sided 95% confidence intervals (CIs) were calculated when appropriate.

Descriptive statistics using the full analysis set (FAS), which included all patients who fulfilled all inclusion criteria, was employed, without data imputation. All results reported are based on the number of FAS patients, unless otherwise specified.

The sample size calculation of the global study was based on standard deviation information on Global Satisfaction measured by TSQM v1.4. A sample size of n=1500 was expected to be able to provide a 95% CI with a half width of 1.01 in the overall study population.^15,34^ For country-specific analysis, it has been estimated that a sample of n=30 – 200 will be able to provide a 95% CI with a half width of 7.47 to 2.79.

Subgroup comparisons of patients with or without any comorbidities were conducted to identify any differences in PMPI, expectations and PROs. For continuous variables, Wilcoxon-Mann-Whitney tests used; for categorical variables, chi-squared tests or exact Fisher tests were used.

## RESULTS

### Clinical parameters and sociodemographic characteristics

A total of 121 patients were enrolled in SENSE study in Greece and were included in the full analysis set (FAS). Demographic characteristics, employment status, and level of education are described in **Table 1**. The patients had mean (SD) age 64.8 (13.9) years (range, 23–90 years) and were predominantly female (82.6%). In total, 16.5 % of the patients were employed full-time, and 57% were retired. RA was shown to have an effect on patient worklife: 4.1% had retired early due to RA-related factors and 4.2% were unemployed or part-time employed.

The patients had established moderate to severe disease (**Table 2**) at the time of recruitment, with a mean (SD) DAS28– erythrocyte sedimentation rate (DAS28-ESR) 4.5 (1.0) and Clinical Disease Activity Index (CDAI) 20.3 (10.1).

Most patients (86.8%) reported ≥1 comorbidity (**Table 3**). The mean (SD) number of comorbidities was 2.7 (2.1), with the most frequent being cardiovascular comorbidities (55.4%) followed by metabolic and nutrition disorders (43%), endocrine disorders (34.7%), musculoskeletal and connective tissue diseases (24.8%), and psychiatric disorders (24.8%).

**Table 3.** Current medications administered for rheumatoid arthritis.

**Current comorbidities, n (%**)	**Full analysis set**
Any	105 (86.8)
Metabolism and nutrition disorders	52 (43.0)
Cardiac disorders	47 (38.8)
Endocrine disorders	42 (34.7)
Musculoskeletal and connective tissue disorders	30 (24.8)
Psychiatric disorders	30 (24.8)
Vascular disorders	25 (20.7)
Gastrointestinal disorders	20 (16.5)
Nervous system disorders	20 (16.5)
Blood and lymphatic system disorders	15 (12.4)
Renal and urinary disorders	13 (10.7)
Respiratory, thoracic and mediastinal disorders	10 (8.3)
Eye disorders	6 (5.0)
Neoplasms benign, malignant and unspecified	4 (3.3)
Infections and infestations	3 (2.5)
General disorders and administration site conditions	2 (1.7)
Hepatobiliary disorders	2 (1.7)
Immune system disorders	2 (1.7)
Skin and subcutaneous tissue disorders	2 (1.7)
Congenital, familial and genetic disorders	1 (0.8)
Ear and labyrinth disorders	1 (0.8)
Reproductive system and breast disorders	1 (0.8)

HRU was high and a previous medical visit for RA was reported by 82.6% of the patients, all of which were on an outpatient basis. The mean (SD) number of visits was from 2.1 (1.1) to 8.5 (4.5) during the previous 3- and 12-month periods prior to enrolment, respectively.

### Medication and Treatment Strategy

The most frequently used RA medications included csDMARDs, namely methotrexate (62.8%), hydroxychloroquine (17.4%), and leflunomide (11.6 %); only 5% of patients were treated with systemic corticosteroids at the time of evaluation (**Table 4**). Among all patients, 43.8% had been treated with bDMARDs; 45.3% of these patients were on monotherapy. Interestingly, despite long-standing disease and suboptimal symptom control with fully tolerable dosages of ongoing DMARD administered for ≥3 months, a switch to a different DMARD was planned by the treating rheumatologist for only half of the patients (49.6%). In 97% of the cases, a bDMARD or tsDMARD was considered as the next step in treatment (most often a tumour necrosis factor inhibitor).

**Table 4.** Medications administered for rheumatoid arthritis.

**RA medication, n (%)**	**Full analysis set**
Methotrexate	76 (62.8)
Other	25 (20.7)
Hydroxychloroquine	21 (17.4)
Leflunomide	14 (11.6)
Infliximab	12 (9.9)
Etanercept	11 (9.1)
Tocilizumab	10 (8.3)
Abatacept	5 (4.1)
Adalimumab	5 (4.1)
Golimumab	5 (4.1)
Rituximab	3 (2.5)
Tofacitinib	2 (1.7)
Certolizumab pegol	1 (0.8)

An analysis of patient medical history showed that 82.6% of patients received treatment for comorbid diseases. The mean (SD) number of medications administered for concurrent diseases was 2.1 (1.9), the most frequent of which were 3-hydroxy-3-methylglutaryl-coenzyme A reductase inhibitors (32.2%), angiotensin-converting enzyme inhibitors (23.1%), thyroid hormones (21.5%), selective beta-blocking agents (19.8%), proton pump inhibitors (16.5%), and selective serotonin reuptake inhibitors (11.6%) or other antidepressants (9.9%) (**Supplementary Table 3**).

### Primary Outcome: Treatment satisfaction

**Figure 1.**
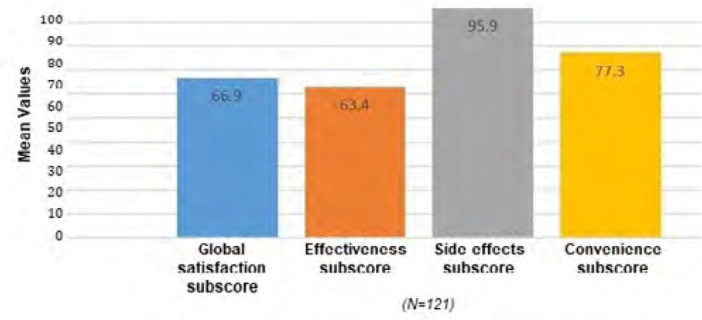
Patients’ satisfaction with RA treatment assessed using the Treatment Satisfaction Questionnaire for Medication version 1.4 Global Satisfaction, Effectiveness, Side Effects, and Convenience domain subscores. RA, rheumatoid arthritis.

The mean (SD) TSQM v1.4 domain scores were as follows: Global Satisfaction, 66.9 (22.4); Effectiveness, 63.4 (21.1); Side Effects, 95.9 (15.4); and Convenience, 77.3 (18.9). The low level of satisfaction was driven by the low effectiveness subs core, in alignment with the suboptimally controlled, active RA (**Figure 1**).

### Secondary Outcomes

RA affected productivity, functional status and overall QoL (**Table 5**). Good self-perceived adherence, defined as ≥80% self-reported adherence, was reported by 93.4% of patients.

**Table 5.** Patient-reported outcomes.

**Parameter, Score Range**	**Patients, n**	**Mean (SD)**
FACIT-F, 0–52	121	30.3 (11.5)
Worst joint pain, 0–10, VAS	121	4.5 (2.8)
Severity of morning stiffness, 0–10, VAS	121	3.6 (3.0)
Duration of morning stiffness, hours^b^	88	1.1 (3.0)
HAQ-DI, 0–3	121	1.1 (0.7)
SF-36 PCS, 100–0	121	39.9 (8.3)
SF-36 MCS, 100–0	121	43.4 (11.1)
WPAI-RA: Presenteeism, %	21	41.0 (25.7)
WPAI-RA: Absenteeism, %	21	2.6 (4.9)
WPAI-RA: Total work productivity impairment, %	21	41.9 (53.9)
WPAI-RA: Total activity impairment, %	21	48.1 (24.5)

FACIT-F, Functional Assessment of Chronic Illness Therapy–Fatigue; HAQ-DI, Health Assessment Questionnaire–Disability Index; MCS, Mental Component Summary; PCS, Physical Component Summary; PGA, Physician Global Assessment of Disease Activity; PtGA, Patient Global Assessment of Disease Activity; RA, rheumatoid arthritis; SF-36, Short-Form, 36-item Health Survey; VAS, visual analogue scale; WPAI-RA, Work Productivity and Activity Impairment–Rheumatoid Arthritis.

### Patient Medication Preference Questionnaire

PMPI questionnaire revealed a preference for oral administration (65.3%) at preferred administration frequencies of once per day (37.2%) or once per week (32.2%). Those preferring parenteral administration showed a preference for biweekly (25.6%) or monthly (40.5%) administration. Notably, 33.1% of patients did not prefer to receive drug combinations. The preferred time to therapeutic effect onset was “up to one week” (ie, the shortest option of the questionnaire) for 52.9 % of patients. The most acceptable adverse events were injection site reaction (21%), deterioration of laboratory values (18.5%), effect on fertility (13.4%), and weight gain (10.9%). Events reported as least acceptable were hair thinning or loss (5.0%), increased risk for cardiovascular diseases (5.9%), allergic reaction (6.7%), and increased risk for malignancies (8.4%).

### Patient Expectations for Pharmacological Treatment

**Figure 2.**
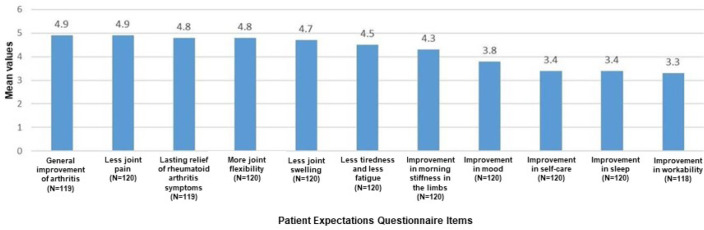
Patients’ expectations for pharmacologic treatment of RA assessed using an 11-item questionnaire.a RA, rheumatoid arthritis. aQuestionnaire used a 7-point rating scale: 1 = no improvement needed, 7 = the most improvement needed.

The highest-rated treatment expectations were general improvement of arthritis, less joint pain, lasting relief of RA symptoms, more joint flexibility, and less joint swelling (**Figure 2**).

### PSP participation

In terms of need for patient support, patients assigned the greatest importance to having access to educational material that focused on RA disease and therapy as well as to a call centre and a starter pack with all information about the patient-support programs.

### Digital health literacy

Based on eHEALS score, the majority of patients were found to have poor familiarity with digital tools for the management of their disease. The mean (SD) patient score was 15.4 (8.8), and the highest patient score was 32. In general, more than half of the participants disagreed or strongly disagreed with the following statements: “I have knowledge of the health resources that are available on the internet”, “I know where and how to find helpful health resources”, “I know how to use the internet to answer my health questions”, and “I know how to use the health information found on the internet”. Only 12.8 % of patients of the 109 available responses agreed or strongly agreed that they can differentiate high-quality from low-quality healthcare resources on the internet, with 6.4% of patients agreed or strongly agreed that they felt confident in using information from the internet to make healthcare decisions.

### Subgroup analysis

Subgroup analysis of PROs between patients with (n=105) versus without (n=16) comorbidities demonstrated that the presence of a comorbid disease correlated statistically significantly with worse physical function (mean [SD] HAQ-DI score 1.0 [0.7] vs 0.6 [0.5] respectively, p=0.001) and lower treatment satisfaction (TSQM Global Satisfaction score 65.5 [22.7] vs 75.9 [18.6] respectively, p=0.049).

Comorbidities were also associated with higher patient expectation for “general improvement of arthritis” (5.1 [1.8] vs 3.9 [1.8], p=0.019), “less joint pain” (5.1 [1.8] vs 4.2 [1.8], p=0.044), “lasting relief of RA symptoms” (5.0 [2.0] vs 3.7 [2.2], p=0.018). The presence of comorbidities was also associated with lower DHL (total eHEALS score 14.5 [8.6] vs 21.6 [7.5], p=0.004).

## DISCUSSION

This study aimed to assess the real-world perspective and treatment expectations of patients with suboptimally controlled RA, information that is considerably underrepresented in the literature. It has been previously shown that patients’ and physicians’ perceptions of RA-related treatment priorities and disease activity may differ.^16,44,45^ The main findings of this subanalysis from the Greek RA patients and the overall SENSE results demonstrated that despite the low level of satisfaction, as determined via TSQM global score, the vast majority of patients had high self-reported adherence to therapy (adherence ≥80%). This finding should be further explored and confirmed in other cohorts. In the Greek subanalysis, both the TSQM global score and good self-reported adherence to treatment slightly exceeded the overall study results. Conversely, to the global SENSE analysis, both the mean TSQM Global Satisfaction and Effectiveness domain subscores were among the lowest, whereas the safety (Side Effects) domain subscore was the highest. The physical function, and overall performance and HRQoL of the patients were negatively affected by the ongoing disease activity. These data further support the concept of the T2T strategy aiming at remission or low disease activity, since better disease’s control might improve patient satisfaction.

Patients’ treatment expectations are associated mainly with control of the disease overall and on specific RA-related symptoms, such as joint pain, swelling, fatigue, and stiffness. Our results suggest a preference for oral versus parenteral therapy among the majority of patients, with about a third not favouring combination therapies, and also a preference for drugs with a rapid onset of action. These results comply with the overall study results. Interestingly, despite suboptimal disease control and long-standing disease, treatment switch to another DMARD was planned by the physicians for only approximately half of the participants in both the global and the present country-specific analysis of SENSE study. The analysis of the global dataset showed that lower patient global satisfaction scores were associated with planned treatment switches.^15,46^ Our country-specific subanalysis did not assess the willingness of patients to receive treatment intensification and, therefore, could not evaluate whether suboptimal patient satisfaction is associated with patient acceptance of treatment changes or vice versa, whether good patient satisfaction despite poor disease control prevents rheumatologists from treatment adjustments. A correlation between therapeutic satisfaction and the patient’s attitude towards treatment has been described,^47,48^ and a recent study has shown that patients who report treatment satisfaction exhibit a weaker inclination to accept treatment intensification, regardless of their DAS28 score and duration of disease.^49^ The data from the SENSE study further corroborate results from other Greek and international studies showing an inconsistency between the treatment recommendations for T2T and clinical practice. The results of a Greek study of patients in the early stages of arthritis similarly showed that only 62.4% of participants who experienced medium or high disease activity after 6 months of treatment were subject to treatment adjustments. The implementation of treatment modifications was reportedly followed by a significant decrease in disease activity after 2 years.^50^ Likewise, in a recent multinational observational study, the T2T guidelines were appropriately applied in only 59% of patient visits.^51^ We believe that the rheumatological community needs to consider carefully these findings to identify the specific barriers of the clinical implementation of T2T concept. Comparable therapeutic inertia, defined as “the failure to initiate or intensify therapy in a timely manner, according to evidence-based clinical guidelines”, is certainly present in the treatment of other chronic diseases.^52^ As literature shows, the potential discordance between physicians and their patients regarding treatment target definition, disease perception and need for treatment adjustment can significantly affect therapeutic decisions in patients with suboptimal disease control, though evaluating the discordance was not the purpose of the study.^53^

Comorbidities in RA are common and have a negative effect on patient functioning, morbidity, and mortality.^4^ Similarly to the overall study results, comorbidities were encountered in the vast majority of the patients from Greece, and 82.6% reported receiving medications for other diseases, with the mean number of drugs administered being 2.1. There was an overlap in the most frequently reported comorbidity categories between the Greek cohort and the overall study population. Nevertheless, except for musculoskeletal/connective tissue disorders, for which the incidence was comparable in the present cohort and overall study population, the incidence of cardiac, metabolic/nutrition disorders, endocrine and psychiatric disorders was higher in the Greek patients. Interestingly, the incidence of psychiatric disorders was 3-fold higher in this subanalysis. It is worth noting that the presence of a comorbid disease correlated with worse disability (HAQ-DI) and lower TSQM global satisfaction scores. These findings further support the importance of the effect of comorbidities on the outcome of RA and the necessity for their effective management. Additionally, patients had poor digital health literacy, and, therefore, poor familiarisation with tools for the management of their disease. Concerning the benefits of digital health resources, the patients reported that their highest prioritization was for receiving information on general RA disease- and medication-related topics through a PSP program and their lowest for digital lifestyle interventions, such as social media, smartphone, and website contents. The eHEALS study results revealed low DHL, highlighting the need to develop health promotional programs addressing DHL and digital tools tailored to the needs and pragmatic capabilities of the RA population. New information- and communication-technologies may substantially contribute in a more accurate monitoring of disease-related parameters while offering much-needed patient education.^54^ As the RA population gradually shifts towards patients with a higher degree of familiarity with digital content and applications, these educational activities could be further developed and applied to a larger group of patients.

Except for the prevalence of females over males, there were differences in the sociodemographic characteristics of this subanalysis and the global SENSE results. Some of these differences, particularly in occupational status, are attributed to the age range of the participants. Thus, based on mean age, the Greek cohort patients were slightly older (mean age of overall study patients 58.4 years old), which in turn accounts for the higher percentage of retired patients in this subanalysis. The observed differences in the incidence of comorbidities between this subanalysis and the overall SENSE results are likely to be attributed to the older age of the current patients. A comparable percentage of patients in this subanalysis and the overall SENSE results had university education. Psychosocial factors, such as education and occupational status as well as demographics, amongst other factors, are likely to influence and account for the potential differences, albeit small, in patient expectations and preferences, DHL and PROs in this subanalysis and the overall SENSE results.

Similarly to this subanalysis, csDMARDs were the most frequently prescribed medications. Differences between individual bDMARD prescriptions in this subanalysis and the global SENSE results can be attributed to local therapeutic protocols and potentially reimbursement policies in the participating countries.

Concerning the limitations of the study, by design, noninterventional studies hold certain limitations, such as selection and recall bias and lack of a control group. The focus on a specific patient group with suboptimal disease control may limit the generalizability of our results to all RA patients. Although PROs reflect subjective patient assessments, however, this effect was counterbalanced by the use of validated PRO tools. Similarly, VAS for the determination of self-reported treatment adherence is validated and highly correlates with electronic monitoring results in patients with chronic conditions, including RA.^40,41,55^ No validated questionnaires were available for assessing the need for PSP, treatment preferences, and expectations. The imbalance in the sizes of groups with and without comorbidities as well as the presence of potential confounding factors warrant caution when interpreting the results of subgroup analysis. These results, therefore, need to be confirmed by using validated measures in future studies. Because of the size of the Greek sample, further subanalyses and correlations to specific outcomes were not possible.

This study provides an in-depth understanding of patient needs and perspectives, also identifying unmet requirements for treatment adjustments that will align with recent therapeutic standards and the T2T principles. Attaining T2T goals under routine clinical practice conditions is increasingly investigated in RA patients. In this context, a longitudinal real-life study in Greece demonstrated that the use of glucocorticoids or ≥2 bDMARDs versus no bDMARDs negatively correlated with low disease activity.^56^ In the aforementioned study, younger age, lower HAQ, body mass index and co-morbidity index were negative predictors of low disease activity, whereas male sex was a positive predictor.

Concluding, the herein presented data showed that RA patients with suboptimal disease control under treatment have low treatment satisfaction and compromised self-reported outcomes, albeit a high self-reported treatment adherence. These data further support both the value of treatment approaches targeting to abrogation of inflammation and emphasise the need of documenting patients’ perspectives to improve disease outcomes.

## AUTHOR CONTRIBUTIONS

All participating authors contributed equally to the gathering of information and writing and reviewing of the article.

## Figures and Tables

**Supplementary Table 1.** List of local ethics committees that provided ethics approval for the SENSE study.

**Site ID**	**Country**	**Primary institution**	**City and postal code**	**Ethics committee**	**Notes**
AR-03	Argentina	DIM Clinica Privada	Ramos Mejía, 1704	Comité de Etica en Investigacion DIM Clinica Privada	—
AR-04	Argentina	Hospital Gral. de Agudos J.M. Ramos Mejía	Buenos Aires, C1221ADC	Comité de Etica en Investigacion Hospital de Agudos J.M. Ramos Mejía	—
AR-05	Argentina	Instituto de Rehabilitación Psicofísica	Buenos Aires, 1428	Comité de Etica en Investigacion Instituto de Rehabilitación Psicofísica (IREP)	—
AR-01	Argentina	CEIM Investigaciones Medica	Buenos Aires, 1425	Comité Independiente de Ética para Ensayos en Farmacología Clínica	—
AR-02	Argentina	Hospital Interzonal Gral Agudos San Martin	La Plata, 1900	Comité de Etica Centro Medico Framingham	—
BR-01	Brazil	Centro Multidisciplinar de Estudos Clínicos	Santo André, BR-CE, 09190-615	Comitê de Ética em Pesquisa da Faculdade de Medicina do ABC (CEP-FMABC)	—
BR-02	Brazil	Santa Casa de Belo Horizonte	Belo Horizante, BR-MG, 30150-221	Comitê de Ética em Pesquisa da Santa Casa de Belo Horizonte (CEP – SCBH)	—
BR-03	Brazil	Fundacao Faculdade Regional de Medicina de São José do Rio Preto	São José Do Rio Preto, BR-CE, 15090-000	Comitê de Ética em Pesquisa em Seres Humanos da Faculdade de Medicina de São José do Rio Preto (CEP-FAMERP)	—
BR-04	Brazil	Centro Mineiro de Pesquisa	Juiz De Fora, BR-MG, 36010570	Comitê de Ética em Pesquisa do Hospital Universitário da Universidade Federal de Juiz de Fora (HU-UFJF)	—
BG-01	Bulgaria	UMHAT Sveti Ivan Rilski	Sofia, 1612	Not required	—
BG-02	Bulgaria	Excelsior Medical Center	Sofia, 1407	Not required	—
CL-02	Chile	Hospital Victoria	Victoria, 4720 000	Comité de Etica de la Investigacion Servicio de Salud Metropolitano Norte	—
CL-01	Chile	Centro Medico Prosalud	Santiago, 7510047	Comité de Etica Cientifica Servicio Salud Araucanía Sur	—
HR-05	Croatia	Klinički Bolnički Centar Split	Split, 21000	Klinički Bolnički Centar Split	The SENSE study was first submitted to the central EC. Based on the submitted documentation, the central EC issued an opinion on the acceptability of the study. After obtaining a positive opinion from the central EC, the clinical trial was submitted to the Agency for Medicinal Products and Medical Devices. Based on the submitted documentation and the central EC’s positive opinion, the Agency for Medicinal Products and Medical Devices granted approval for study conduct. Some institutions (hospitals) also requested that the study be submitted to their Institutional Committees, so approvals were also obtained from the Institutional Committees in Croatia listed in column E.
HR-01	Croatia	Klinički Bolnički Centar Zagreb	Zagreb, 10000	Klinički Bolnički Centar Zagreb	
HR-03	Croatia	Klinički Bolnički Dubrava Zagreb	Zagreb, 10000	Klinički Bolnički Dubrava Zagreb Klinička Bolnica Dubrava Zagreb	
HR-04	Croatia	Klinički Bolnički Centar Sestre Milosrdnice	Zagreb, 10000	Klinički Bolnički Centar Sestre Milosrdnice	
HR-02	Croatia	Klinički Bolnički Centar Zagreb	Zagreb, 10000	Klinički Bolnički Centar Zagreb	
CZ-02	Czech Republic	Revmatolog s.r.o.	Jihlava, 58601	Ethics Committee of the University Hospital Motol, V Úvalu 84, Prague 5, 150 06, Czech Republic	—
CZ-04	Czech Republic	Revma Praha s.r.o.	Prague, 15800	Ethics Committee of the University Hospital Motol, V Úvalu 84, Prague 5, 150 06, Czech Republic	—
CZ-06	Czech Republic	Fakultni Nemocnice v Motole	Prague, 15006	Ethics Committee of the University Hospital Motol, V Úvalu 84, Prague 5, 150 06, Czech Republic	—
CZ-01	Czech Republic	INREA s.r.o.	Ostrava, 703 00	Ethics Committee of the University Hospital Motol, V Úvalu 84, Prague 5, 150 06, Czech Republic	—
CZ-05	Czech Republic	Revmatologicke Centrum s.r.o.	Velke Bilovice, 69102	Ethics Committee of the University Hospital Motol, V Úvalu 84, Prague 5, 150 06, Czech Republic	—
CZ-03	Czech Republic	Revimex PRO s.r.o.	Karvina, 733 01	Ethics Committee of the University Hospital Motol, V Úvalu 84, Prague 5, 150 06, Czech Republic	—
EE-02	Estonia	Tartu University Hospital	Tartu, 50406	Research Ethics Committee of the National Institute for Health Development	—
EE-01	Estonia	East Tallinn Central Hospital	Tallinn, 11312	Research Ethics Committee of the National Institute for Health Development	—
EE-03	Estonia	Pärnu Hospital	Tallinn, 11312	Research Ethics Committee of the National Institute for Health Development	—
GR-01	Greece	University General Hospital of Heraklion, Crete	Voutes Herakleio, 71500	IRB/IEC of the University General Hospital of Heraklion, Crete	—
GR-02	Greece	Metropolitan General Hospital	Athens, 15562	IRB/IEC of the Metropolitan General Hospital	—
GR-03	Greece	OLYMPION Hospital – General Clinic of Patras	Patras, 26221	IRB/IEC of the OLYMPION Hospital – General Clinic of Patras	—
GR-04	Greece	IASIO-General Clinic of Kallithea	Kifissia, 14561	IRB/IEC of the IASIO-General Clinic of Kallithea	—
GR-05	Greece	University General Hospital of Alexandroupoli	Alexandroupoli, 68100	IRB/IEC of the University General Hospital of Alexandroupoli	—
GR-06	Greece	Euromedica General Clinic of Thessaloniki	Thessaloniki, 54623	IRB/IEC of the Euromedica General Clinic of Thessaloniki	—
GR-07	Greece	Henry Dunant Hospital Center	Athens, 11526	IRB/IEC of the Henry Dunant Hospital Center	—
GR-08	Greece	General Hospital of Patras «Agios Andreas»	Patras, 26335	IRB/IEC of the General Hospital of Patras «Agios Andreas»	—
GR-09	Greece	Naval Hospital of Athens	Athens, 11521	IRB/IEC of the Naval Hospital of Athens	—
HU-01	Hungary	Budai Irgalmasrendi Kórház	Budapest, 1027	Study protocol approval was obtained from the central EC: the Medical Research Council, Scientific and Research Ethics Committee, Hungary	—
HU-03	Hungary	Békés Megyei Pándy Kálmán Kórháza	Gyula, 5700		
HU-04	Hungary	Hévízgyógyfürdő és Szent András Reumakórház	Heviz, 8380		
HU-06	Hungary	Szabolcs – Szatmár – Bereg Megyei Kórházak és Egyetemi Oktató Kórház	Nyiregyhaza, 4400		
HU-05	Hungary	Miskolci Semmelweis Kórház és Egyetemi Oktatókórház	Miskolc, 3529		
HU-02	Hungary	Petz Aladár Megyei Oktató Kórház	Gyor, 9023		
IR-03	Ireland	St. James’s Hospital	Dublin 8,00000	Tallaght University Hospital/St. James’s Hospital Joint Research Ethics Committee. Tallaght University Hospital, Dublin 24, Ireland	—
IR-02	Ireland	Cork University Hospital	Cork, T12 DFK4	Clinical Research Ethics Committee of the Cork Teaching Hospitals, Lancaster Hall, 6 Little Hanover Street, Cork, Ireland	—
IR-01	Ireland	Croom Orthopaedic Hospital	Limerick, V35 F434	HSE Mid-Western Regional Hospital Research Ethics Committee, University Hospital Limerick, Limerick, Ireland	—
JP-08	Japan	Nagasaki University	Nagasaki, 852-8501	長崎大学病院臨床研究倫理 委員会 (Nagasaki University Hospital Clinical Research Ethics Committee)	—
JP-06	Japan	Kyoto Prefectural University of Medicine	Kyoto-Shi, 602-8566	京都府立医科大学医学倫理審査委員会 (Kyoto Prefectural University of Medicine Medical Ethics Review Committee)	—
JP-05	Japan	Kobe University	Kobe-Shi, 650-0017	神戸大学医学部附属病院臨床研究推進センタ ー倫理審査委員会 (Kobe University Hospital Clinical & Translational Research Center)	—
JP-09	Japan	Yoshida Orthopaedic Clinic	Morioka, 020-0015	代々木メンタルクリニッ ク倫理審査委員会 (Yoyogi Mental Clinic Ethical Review Committee)	—
JP-02	Japan	Setagaya Rheumatology Clinic	Tokyo, 156-0052	代々木メンタルクリニック倫理審査委員会 (Yoyogi Mental Clinic Ethical Review Committee)	—
JP-03	Japan	Hiroshima University	Hiroshima-Shi, 734-8551	広島臨床研究開発支援センター臨床研究倫理審査委員 会 (Clinical Research Center in Hiroshima)	—
JP-07	Japan	Hokkaido University	Sapporo-Shi, 060-8648	北海道大学病院自主臨床研究審査委員会 (Hokkaido University Hospital Division of Clinical Research Administration)	—
JP-01	Japan	Yamagata University School of Medicine	Yamagata-Shi, 990-9585	山形大学医学部倫理審査委員会 (Ethical Review Committee of Yamagata University Faculty of Medicine)	—
JP-04	Japan	The University of Tokyo	Tokyo, 113-8655	東京大学大学院医学系研究科・医学部 介入等研究倫理委員会 (Graduate School of Medicine and Faculty of Medicine, the University of Tokyo)	—
LV-01	Latvia	P. Stradins Clinical University Hospital	Riga, 1002	Ethics Committee for Clinical Research at Pauls Stradins Clinical University Hospital Development Society	—
LT-02	Lithuania	Hospital of Lithuanian University of Health Sciences Kaunas Clinics	Kaunas, 50161	Kaunas Regional Biomedical Research Ethics Committee	—
LT-01	Lithuania	Klaipeda University Hospital	Klaipeda, 92288	Kaunas Regional Biomedical Research Ethics Committee	—
PL-05	Poland	Slaskie Centrum Reumatologii	Ustroń, 43-450	EC not required – notification processed	—
PL-02	Poland	Specjalistyczna Praktyka Lekarska Katarzyna Smolik	Tychy, 43-100	EC not required – notification processed	—
PL-04	Poland	Ortopedyczno-Rehabilitacyjny Szpital Kliniczny	Poznan, 61-545	EC not required – notification processed	—
PL-03	Poland	Gabinet Internistyczno – Reumatologiczny Izabela Domyslawska	Bialystok, 15-276	EC not required – notification processed	—
PL-01	Poland	Prywatny Gabinet Lekarski – Grazyna Swierkowska	Lodz, 93-513	EC not required – notification processed	—
RO-07	Romania	Spitalul Clinic Dr. I. Cantacuzino	Bucharest, 020475	National Committee of Bioethics for Medicines and Medical Devices	—
RO-01	Romania	Spitalul Clinic Sfanta Maria Bucuresti	Bucharest, 011172	National Committee of Bioethics for Medicines and Medical Devices	—
RO-02	Romania	Spitalul Clinic Sfanta Maria Bucuresti	Bucharest, 011172	National Committee of Bioethics for Medicines and Medical Devices	—
RO-03	Romania	Spitalul Clinic de Recuperare Iasi	Iasi, 700661	National Committee of Bioethics for Medicines and Medical Devices	—
RO-08	Romania	Spitalul Clinic de Recuperare Iasi	Iasi, 700661	National Committee of Bioethics for Medicines and Medical Devices	—
RO-04	Romania	Spitalul Clinic Judetean de Urgenta Cluj	Cluj-Napoca, 400006	National Committee of Bioethics for Medicines and Medical Devices	—
RO-05	Romania	Spitalul Clinic de Recuperare Cluj-Napoca	Cluj-Napoca, 400437	National Committee of Bioethics for Medicines and Medical Devices	—
RO-06	Romania	Spitalul Clinic Judetean de Urgenta Targu Mures	Targu Mures, 540136	National Committee of Bioethics for Medicines and Medical Devices	—
RU-03	Russia	Institution KhMAO-Ugra Regional Clinical Hospital	Khanty-Mansiysk, 628011	Independent Interdisciplinary Ethics Committee for Clinical Studies	—
RU-02	Russia	Research Institute of Rheumatology	Moscow, 115522	Independent Interdisciplinary Ethics Committee for Clinical Studies	—
RU-01	Russia	Moscow Regional Research Clinical Institute MF Vladimirskiy	Moscow, 129110	Independent Interdisciplinary Ethics Committee for Clinical Studies	—
RU-04	Russia	Yaroslavl State Medical University	Yaroslavi, 150000	Independent Interdisciplinary Ethics Committee for Clinical Studies	—
SK-02	Slovakia	ROMJAN s.r.o.	Bratislava, 821 08	Ethics Committee of Bratislava Autonomous Region, Sabinovská 16, 820 05 Bratislava, Slovak Republic	—
SK-03	Slovakia	Novamed s.r.o.	Banská Bystrica, 97405	Independent Ethics Committee of Banská Bystrica Autonomous Region, Nám. SNP 23, 974 01, Banská Bystrica, Slovak Republic	—
SK-04	Slovakia	Univerzitna Nemocnica Bratislava	Bratislava, 82606	Ethics Committee, University Hospital Bratislava, Pažítková 4, 821 01 Bratislava, Slovak Republic	—
SK-01	Slovakia	Ambulance Karpatská – Private Practice	Poprad, 058 01	Ethics Committee of Prešov Autonomous Region, Námestie Mieru 2, 080 01 Prešov, Slovak Republic	—
TR-05	Turkey	Inonu University Turgut Ozal Medical Center Education and Research Hospital	Malatya, 44280	One central EC under coordinating site per local regulation	One central EC under coordinating site per local regulation
TR-01	Turkey	Hacettepe University Faculty of Medicine	Ankara, 6100	Hacettepe University Clinical Research Ethic Boards (one central EC under coordinating site per local regulation)	
TR-02	Turkey	Marmara University Istanbul Pendik Education and Research Hospital	Istanbul, 34899	One central EC under coordinating site per local regulation	
TR-03	Turkey	Sivas Cumhuriyet University Health Services Application and Research Hospital	Sivas, 58140	One central EC under coordinating site per local regulation	
TR-04	Turkey	Istanbul University Cerrahpasa-Cerrahpasa Faculty of Medicine	Istanbul, 34098	One central EC under coordinating site per local regulation	One central EC under coordinating site per local regulation
TR-07	Turkey	Osmangazi University Faculty of Medicine	Eskisehir, 26480	One central EC under coordinating site per local regulation	
TR-08	Turkey	Trakya University Faculty of Medicine	Edirne, 22030	One central EC under coordinating site per local regulation	
TR-10	Turkey	Akdeniz University Faculty of Medicine	Antalya, 07070	One central EC under coordinating site per local regulation	
TR-11	Turkey	Adnan Menderes University Faculty of Medicine	Aydin, 09010	One central EC under coordinating site per local regulation	
TR-12	Turkey	Gulhane Education and Research Hospital	Ankara, 06010	One central EC under coordinating site per local regulation	
TR-14	Turkey	Necmettin Erbakan University Meram Faculty of Medicine Hospital	Konya, 42080	One central EC under coordinating site per local regulation	
TR-06	Turkey	Bahcesehir University Hospital Medical Park Goztepe	Istanbul, 34732	One central EC under coordinating site per local regulation	
TR-13	Turkey	Namık Kemal University Faculty of Medicine Application and Research Hospital	Tekirdağ, 59030	One central EC under coordinating site per local regulation	
TR-09	Turkey	Mustafa Kemal University Hospital	Hatay, 31001	One central EC under coordinating site per local regulation	
UY-01	Uruguay	Medica Uruguaya	Montevideo, 11300	Comité de Etica de Medica Uruguaya	—
UY-02	Uruguay	Ascociacion Española Primera de Socorros Mutuos	Montevideo, 11200	Comité de Etica AESM	—

EC, ethics committee.

**Supplementary Table 2.** Questionnaire to assess medication preferences.

We would like to ask you about your preferences regarding medication used for rheumatoid arthritis. For each question, please circle one answer which is most likely to reflect your opinion. What is the preferred route of administration? Parenteral: intravenous Parenteral: subcutaneous Oral What is the preferred frequency of administration in the case of parenteral administration? Biweekly Monthly 3-monthly 6-monthly What is the preferred frequency of administration in the case of oral administration? Twice per day Once per day Once per week What is the preferred time until the effect of onset? Up to 1 week Up to 2 weeks Up to 1 month Up to 3 months What is your preference regarding drug combinations used for your rheumatoid arthritis? Drug combination is not preferred Treatment which requires daily combination is acceptable Treatment which requires combination with another drug once a week is acceptable What is the most acceptable potential side effect of the medication used for rheumatoid arthritis? Increased risk for infections Allergic reaction Deterioration of my laboratory values Increased risk for malignancies Weight gain Hair thinning or loss Skin symptoms, eg, injection site reaction, rash Effect on fertility Increased risk for cardiovascular diseases

**Supplementary Table 3.** Medications for concomitant diseases (≥5% of patients).

Concomitant medications* n (%)	**Full analysis set**
Any concomitant medication	100 (82.6)
C10AA - HMG CoA reductase inhibitors	39 (32.2)
C09CA - Angiotensin II receptor blockers (ARBs), plain	28 (23.1)
H03AA - Thyroid hormones	26 (21.5)
C07AB - Beta blocking agents, selective	24 (19.8)
A02BC - Proton pump inhibitors	20 (16.5)
N06AB - Selective serotonin reuptake inhibitors	14 (11.6)
N06AX - Other antidepressants	12 (9.9)
C03AA - Thiazides, plain	11 (9.1)
N05BA - Benzodiazepine derivatives	11 (9.1)
A10BA - Biguanides	10 (8.3)
C09DA - Angiotensin II receptor blockers (ARBs) and diuretics	10 (8.3)
N03AX - Other antiepileptics	10 (8.3)
A11CC - Vitamin D and analogues	9 (7.4)
C08CA - Dihydropyridine derivatives	8 (6.6)
M05BA - Bisphosphonates	8 (6.6)
B01AC - Platelet aggregation inhibitors excl. heparin	7 (5.8)
A10BD - Combinations of oral blood glucose lowering drugs	6 (5.0)
C05CA - Bioflavonoids	6 (5.0)

* Anatomical Therapeutic Chemical Classification coding level 4

## Abbreviations

bDMARD: Biological DMARD
CDAI: Clinical Disease Activity Index
CI: Confidence interval
CRP: C-reactive protein
DHL: Digital health literacy
csDMARD: Conventional synthetic DMARD
DAS28: Disease Activity Score: 28 joints
DMARD: Disease-modifying anti-rheumatic drug
eHEALS: Electronic Health Literacy Scale
ESR: Erythrocyte sedimentation rate
EULAR: European League Against Rheumatism
FACIT-F: Functional Assessment of Chronic Illness Therapy – Fatigue
FAS: Full-analysis set
HAQ-DI: Health Assessment Questionnaire – Disability Index
HRQoL: Health-related quality of life
NRS: Numerical rating scale
PSP: Patient-support programme
RA: Rheumatoid arthritis
SDAI: Simplified Disease Activity Index
SF-36: Short Form 36 Health Survey Questionnaire
SJC28: Swollen joint count based on a 28-joint assessment
TJC28: Tender joint count based on a 28-joint assessment
tsDMARD: Targeted synthetic DMARD
T2T: Treat-to-target
TSQM: Treatment Satisfaction Questionnaire for Medication
VAS: Visual analogue scale
WPAI-RA: Work Productivity and Activity Impairment -Rheumatoid Arthritis
